# New insights of low-temperature plasma effects on germination of three genotypes of *Arabidopsis thaliana* seeds under osmotic and saline stresses

**DOI:** 10.1038/s41598-019-44927-4

**Published:** 2019-06-17

**Authors:** Maxime Bafoil, Aurélie Le Ru, Nofel Merbahi, Olivier Eichwald, Christophe Dunand, Mohammed Yousfi

**Affiliations:** 1 LAPLACE, UMR CNRS 5213, Université Paul Sabatier, Toulouse, France; 2 LRSV, UMR CNRS 5546, Université Paul Sabatier, Castanet-Tolosan, France; 3 Fédération de Recherche 3450, Plateforme Imagerie, Pôle de Biotechnologie Végétale, Castanet-Tolosan, France

**Keywords:** Salt, Biomedical engineering

## Abstract

In order to investigate the effects of low temperature plasmas on germination of *Arabidopsis thaliana* seeds, a dielectric barrier discharge device generating the plasma in ambient air was used. To highlight the different plasma effects on the seed surface, saline and osmotic stresses were considered in the case of reference Col-0 seeds and two further seed coat mutants *gl2* and *gpat5* to better analyse the seed surface changes and their consequences on germination. The *GL2* gene encode a transcription factor controlling the balance between the biosynthesis of fatty acids in the embryo and the production of mucilage and flavonoid pigments in the seed coat. The *GPAT5* gene encode for an acyltransferase necessary for the accumulation of suberin in the seed coat which is essential for the embryo protection. The testa and endosperm ruptures are identified to note the germination stage. An increasing of germination rate, possibly due to the modification of mantle layers structure, is observed in most of cases, even in presence of saline or osmotic stress, after plasma treatment. Furthermore, we demonstrated that the germination rate of the *gl2* mutant seeds is increased by at most 47% after plasma treatment, contrariwise, the germination of *gpat5* mutant being initially lower is inhibited by the same plasma treatment. The scanning electron microscopy pictures and confocal microscopy fluorescence both showed changes of the exterior aspects of the seeds after plasma treatment. Considering these results, we assumed that lipid compounds can be found on the surface. To validate this hypothesis, permeability tests were performed, and it was clearly shown that a permeability decrease is induced by the low temperature plasma treatment.

## Introduction

Germination is a vital process in plant development. It gathers events that result from the water absorption by the quiescent dry seed and end with the lengthening of the embryonic axis^1^. In order to carry out this essential and crucial step, the environmental conditions of the seeds must be favourable at a given moment and for a sufficient period^2^.

Overall, conditions are reaching their optimum only over a short period of time; the acceleration of the germination rate is a major asset for the survival of a species. The seeds are often treated by many phytosanitary products before sowing^3^. The use of such chemical products has major impacts on both the environment and the human health. For the purpose of reduction of pesticide use, researchers are trying to develop multiple ways to find substitute to these chemicals.

In order to find other substitute methods based more particularly on the use of the low temperature plasmas, the collaboration between plant biology and plasma physics aims to find alternative solutions to increase the germination rate while preserving both the environment and the seed quality.

Low temperature plasmas can be produced by several kinds of electric discharges using different background gas compositions and pressure and electric or electromagnetic power supplies^4^. The resulting weakly ionized gas, composed by low densities of energetic electrons with dissociated, excited and ionized species, is called non-thermal plasmas since there is a strong thermal non-equilibrium between the different species. It is also called low temperature plasmas because the mean plasma temperature generally does not exceed the body temperature (about 37 °C) thus allowing the treatment of living cells without any significant thermal effects.

In fact, the low temperature plasmas are a good candidate in the plant biology field because they firstly showed their efficiency in many biological applications^5^. For many years, the antibacterial properties of low temperature plasma have been used for the sterilization of medical equipment and the decontamination of surfaces^6,7^. Low temperature plasmas are also known for their healing properties^8,9^ or for the induced changes of biological surface properties^10,11^. Low temperature plasmas also contribute to tumour cell reduction^12^ and even can partially permeabilize cell membranes for gene transfection applications^13^.

In the field of plasma agriculture, previous studies have shown that the germination rate of various seeds of agronomic interest such as tomato, mustard, soybean…^14–17^ can be stimulated by the low temperature plasmas. However, only few studies have investigated the mechanisms responsible for the improvement of the seed germination rate and the plant growth after the plasma treatment^18^. Further research studies are therefore needed in order to better explain the processes involved in seed germination and development.

In the present work, the model plant *Arabidopsis thaliana* was chosen because its growth conditions and its short life cycle are favourable to laboratory experiments^19^. Moreover, this plant does not have economic interest, which allows the diffusion and the exchange of results in the scientific community without confidentiality problem. In addition, the results obtained could be easily transferred to close related plants with agronomical interest.

The seed is composed of several external layers, aiming to protect the embryo. The outermost layer is also called the testa and it protects the endosperm. The rupture of all these peripheral layers which occurs sequentially, allows the seed to initiate its first stage of growth: the germination^20^.

In a previous work^21^, it was more particularly shown the good efficiency of the low temperature plasmas on the increase of the germination rate of the *A. thaliana* ecotype Col-0. In order to better understand the effects of the low temperature plasma on early germination steps and also on the seed surface modification, we considered in the present work, two further mutants of *A*. *thaliana* (*gpat5* and *gl2*). The *GL2* (*AT1G79840*) gene encodes for a transcription factor, which control the morphological development of trichomes but also the normal development of the seed coat and mucilage^22^. The seeds of the *gl2* mutant have an altered layer mantle, a reduced mucilage release and a lower germination rate^23^. The GPAT5 (AT3G11430) protein is an acyltransferase necessary for the proper production of fatty acids^24,25^. The *gpat5* mutants are affected in the synthesis of suberin and cutin, which means that seeds of the mutant cannot produce any cuticle that is part of the seed coat which protect the embryo^26^. Interestingly, these two genes have been found to be expressed in the seed outer cell layer during seed formation (Supplementary Fig. S1; Supplementary Tables 1–2)^27^.

In fact, many compounds as reactive oxygen species (ROS) are involved during the germination process^28–30^. ROS are constitutively produced in the plant during photosynthesis, photorespiration and respiration^31^ and have a dose-dependent effect^32^. They are known to be produced in stress condition such as saline stress^33^. At a high concentration, the oxidizing power is important and deleterious for the regular plant development. But at lower concentrations, they could act as secondary messengers^16,34^. In order to maintain a non-deleterious level of ROS, plants possess a battery of proteins able to regulate the ROS homeostasis^35^. Among these proteins, class III peroxidases (CIII Prxs) can reduce or oxidize hydrogen peroxide to regulate its concentration^36^. In the literature, the hydrogen peroxide and the CIII Prxs activity were shown to be colocalized in the seed coat prior the rupture of these two protective layers^32,37^. Some CIII Prxs genes are expressed during the first germination steps (Supplementary Fig. S2), it is thinkable that the protein encoding genes may play a role in the rupture of those layers of the embryo and thereby improving the germination^37^.

Saline stress has negative effects on germination due to ionic imbalances^38^ and interference in the uptake of nutrients essential for the plant development^39^. This could be highlighted, in the presence of a strong concentration of NaCl, by large reduction of germination rate and germination speed of the seeds^40^ (i.e. the treated seeds need less time to have a given germination rate than the control one). As NaCl has both saline and osmotic effects, osmotic stress alone can be tested with the use of Polyethylene Glycol (PEG). It was found to have a comparable effect to heavy metals and interferes with the water transport within plants^41^. The consequence is the necrosis of adult plant. If the seed is treated with PEG during germination a variable reduction and delay in the process will be observed^42^.

Therefore, in the following we will analyze the effects of the low temperature plasma generated in ambient air using a floating electrode dielectric barrier discharge (FE-DBD) setup on the seed germination and surface under saline and osmotic stresses in the case of three *A. thaliana* genotypes seeds (Col-0, *gl2* and *gpat5* mutants). Col-0 is the wild type seed, it carries the functional genes *GL2* and *GPAT5*.

## Results

### Effects of plasma treatments under saline and osmotic stresses

**Figure 1:**
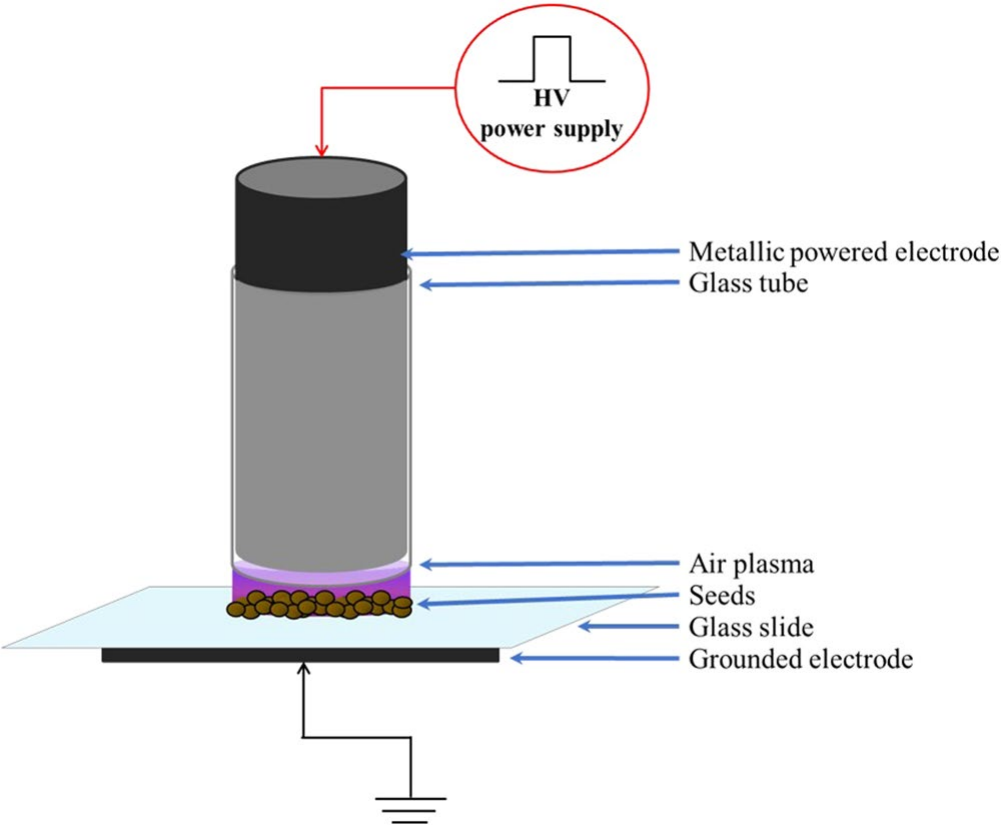
Scheme of the plasma device of dielectric barrier discharges (DBD) in ambient air. Interior glass tube diameter: 8 mm; glass slide thickness: 1 mm; metallic powered electrode diameter: ~8 mm.

**Figure 2:**
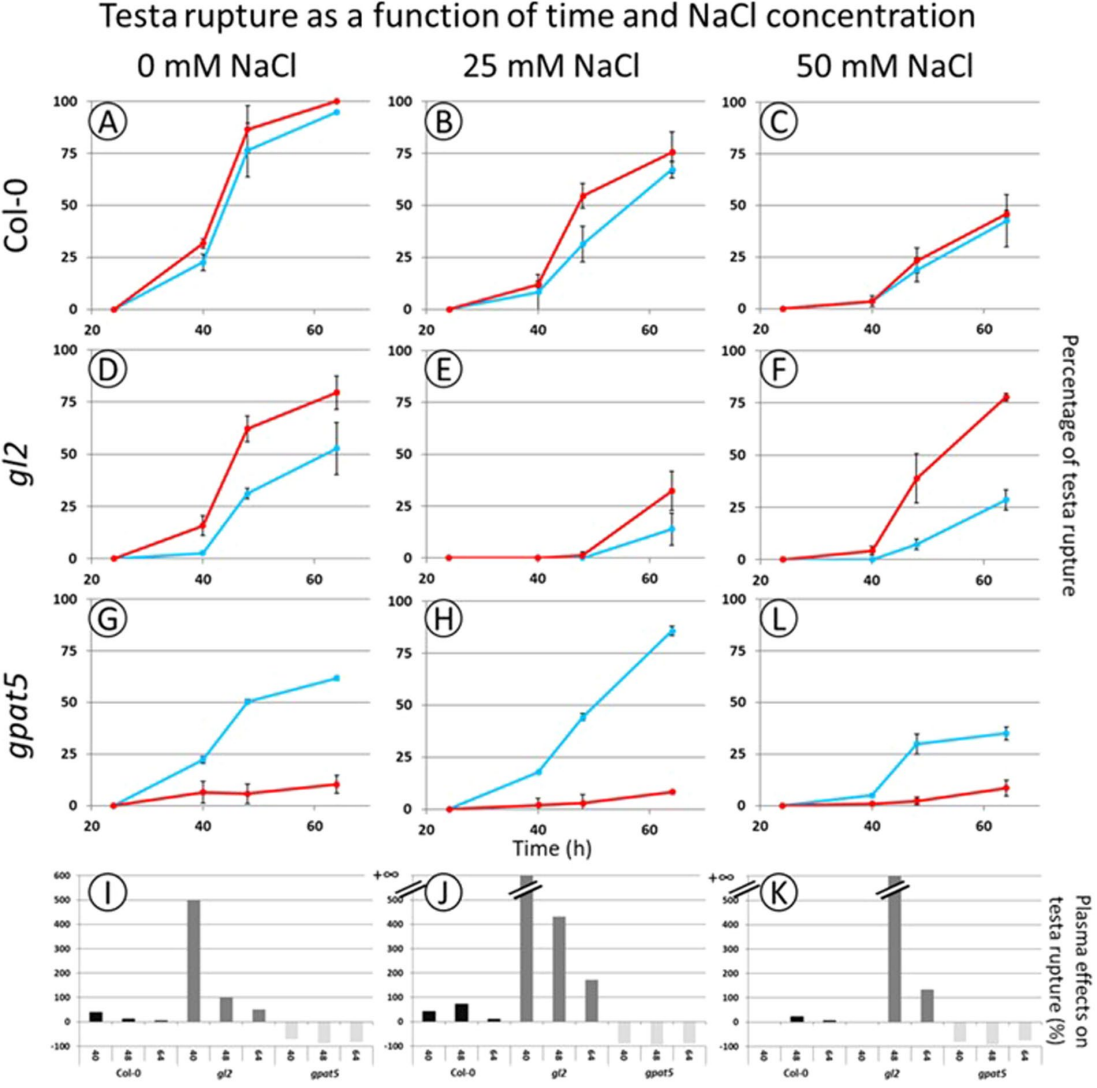
Plasma treatment has opposite effect on germination of *A. thaliana* Col-0*, gl2* and *gpat5* seeds under saline condition. This means that Col-0 and gl2 seeds showed a plasma stimulation of the germination rate while gpat5 showed the inverse effect (i.e. an inhibition of the germination). The testa rupture was evaluated as a function of time and different genotypes. The blue curves represent the control seeds and the red the plasma treated seeds. (**A**–**C)**: Col-0; (**D**–**F)**: *gl2*; (**G**–**I)**: *gpat5*. 3 different germination conditions were tested: control without NaCl (**A**,**D**,**G**,**J**); 25 mM of NaCl (**B**,**E**,**H**,**K**); 50 mM of NaCl (**C**,**F**,**I**,**L**). Due to the lack of germination with NaCl concentration higher than 50 mM, the obtained percentage of testa rupture are not shown. The bar charts represent the plasma effect on the testa rupture compared to the control one (**J**–**L**).

In the context of global climate change, the effect of low temperature plasma on seeds germination under salt or osmotic stresses is very interesting to analyze. For the seeds treatment we used the low temperature plasma DBD device displayed in Fig. 1 and described in material and method section. First of all, in standard growth conditions without any specific stress, the low temperature plasma treatment increases the speed of testa and endosperm ruptures of the ecotype Col-0 of *A. thaliana* (Fig. 2A). However, the rate of germination was not affected. Indeed, after 64 hours of imbibition, the control curve catches up the plasma treated curve in the case of the testa rupture (TR) when the maximum of ruptures is reached (between 95 and 100%).

In addition, our previous studies have shown an effect of the low temperature plasma on the seed surface of the genotype Col-0^21^. It is therefore of interest to determine if these differences are still observable on the two other considered genotypes. As already underlined, the mutants *gl2* and *gpat5* have been chosen because they have abnormal seed coat layers. The germination of the two different mutants of *A. thaliana* was analyzed and compared with Col-0 (Fig. 2). The germination rate and speed of *gl2* and *gpat5* seeds are considerably reduced compared to that of Col-0. After 64 h, it is reduced from 95% for Col-0 down to 50% for *gl2* and *gpat5 seeds*. Despite this, the plasma treatment increases the germination for the *gl2* seeds (Fig. 2D) but prevents the germination of the *gpat5* seeds by inhibiting the testa rupture (Fig. 2G).

In saline stress growth condition, the plasma stimulation of the seed germination was always observable since the plasma treated seeds have a better germination rate than the control seeds for Col-0 and *gl2* (Fig. 2B,C,E,F). However, the germination rate of the plasma treated seeds is lower than the non-stressed case and decreases with the increasing concentration of NaCl (from 0 to 50 mM). It is noteworthy that germination is totally inhibited from 100 mM NaCl (data not shown). Therefore, the low temperature plasma treatment partially restores the germination rate that decreased under saline stress condition. 64 h after sowing with 25 mM NaCl, the plasma treated seeds reach a rate of 78% of TR whereas the non-treated seeds are only at 29% of TR for *gl2* seeds (Fig. 2E).

The bar charts display the plasma effects on the percentage of the testa rupture compared to the control seeds (Fig. 2J–L). As we can see, the negative effect (i.e. inhibition of TR) of the plasma on the testa rupture of *gpat5* mutant seeds is constant. Moreover, the positive effect on Col-0 seeds (i.e. increase of TR proportion) becomes lower with the augmentation of the salt concentration. Then, we must note that the positive effect of the plasma is more important for the *gl2* mutant seeds but that this effect decreases over time since the non-treated seeds catch up the delay.

**Figure 3:**
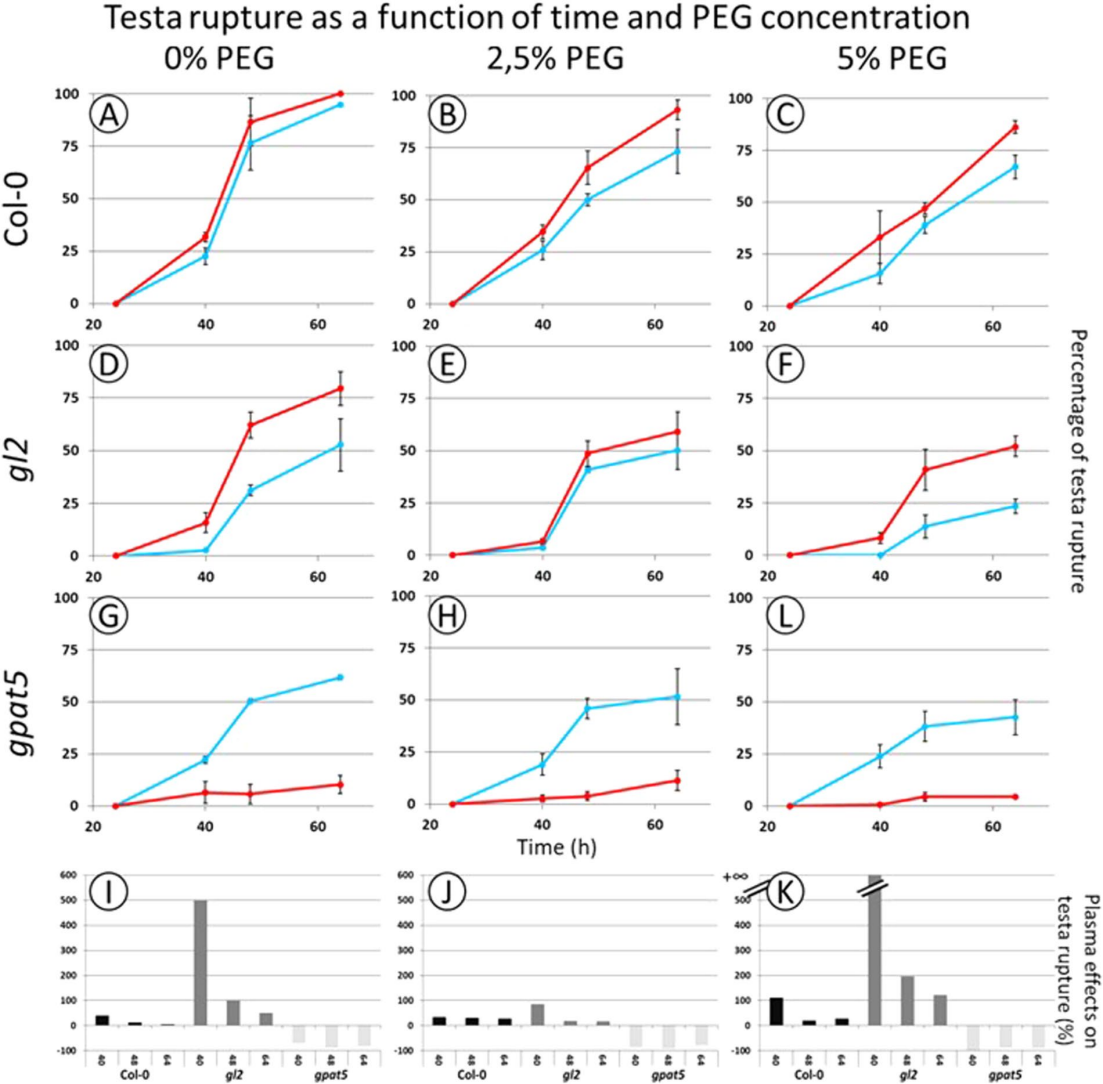
Plasma treatment has opposite effect on germination of *A. thaliana* Col-0*, gl2* and *gpat5* seeds under osmotic condition. This means that Col-0 and gl2 seeds showed a plasma stimulation of the germination rate while gpat5 showed the inverse effect (i.e. an inhibition of the germination). The testa rupture was evaluated as a function of time and different genotypes. The blue curves represent the control seeds and the red the plasma treated seeds. (**A**–**C)**: Col-0; (**D**–**F)**: *gl2*; (**G**–**I)**: *gpat5*. 3 different germination conditions were tested: control without PEG (**A**,**D**,**G**,**J**); 2,5% of PEG (**B**,**E**,**H**,**K**); 5% of PEG (**C**,**F**,**I**,**L**). Due to the lack of germination with PEG content higher than 5%, the obtained percentage of testa rupture are not shown. The bar charts represent the plasma effect on the testa rupture compared to the control one (**J**–**L**).

In order to separate the effects of osmotic and saline stresses, *A. thaliana* seeds were germinated in presence of various PEG 6000 percentages. Increasing the percentage of PEG has a detrimental impact on the germination rate (Fig. 3). With 15% PEG, both control and plasma treated seeds did not germinate (data not shown). As observed in the case of NaCl stress, the treatment of seeds with plasma partially restores the germination rate for the seed grown on 5% PEG for Col-0 and *gl2* seeds (Fig. 3C,F). Using the literature, an equivalent osmotic pressure expressed in bar could be calculated^43,44^ (Supplementary Fig. S2), thus showing that 25 mM of NaCl is equivalent to 10% of PEG. But, the germination at 25 mM of NaCl is higher than the germination observed with 10% of PEG. Then, at equal osmotic pressure the PEG has more inhibitory effect than the NaCl. In parallel, the positive effect of plasma treatment seems to be lower in the case of seeds germinated in presence of PEG (Figs 2, 3).

### Modification of seeds surface

**Figure 4:**
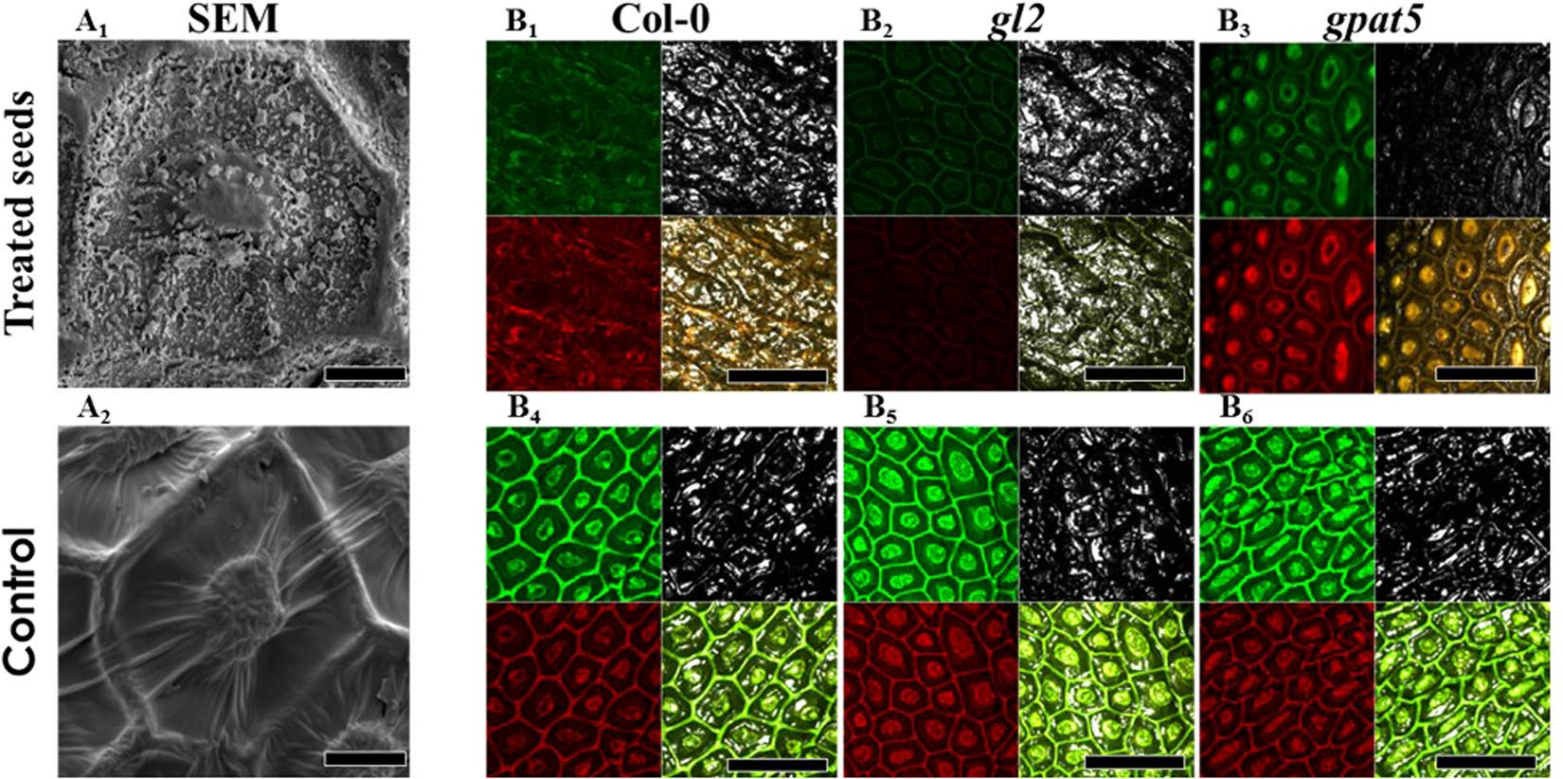
Low temperature plasma affects *A. thaliana* seeds surface. (**A**) Col-0 seed columella with or without plasma treatment observed with a scanning electron microscopy. Scale bar: 10 µm. (**B**) Confocal observation of seed from the three different *A. thaliana* genotypes with or without plasma treatment using lens 10X zoom x9. The seeds are previously stained with auramine-O. After excitation at 488 nm, seed surface was visualized by reflection at 484–494 nm (gray); by fluorescence at 505–560 nm (green), by fluorescence at 572–642 nm (red). The right bottom panel corresponds to the merged of the 3 detection. Scale bar: 100 µm.

Scanning electron microscopy (SEM) pictures show the appearance of particular structure on the treated seed surface (Fig. 4A). There is no significant difference between the ecotype Col-0, and the two mutants *gl2* and *gpat5* for control seeds (Fig. 4A_2_,B_’4_,B_5_,B_6_). In parallel, Auramine-O staining, allows to visualize by fluorescence the hydrophobic compounds such as lignin which is one of the structural compounds of the seed^45,46^. The contours of the cells and the columella are visible on all shots. In the case of the pictures obtained for plasma treated seeds, surface modifications are observed (Fig. 4A_1_,B_1_,B_2_,B_3_). On the reference ecotype Col-0, the fluorescent signal is decreased, the contours of the cells and columella are very sparsely visible in the case of the treated seeds. For the seeds of *gl2 m*utants, there is a very strong loss of the fluorescent signal; nevertheless, the walls and columella remain recognizable. For the *gpat5* mutant, we observe a lower signal loss and even an increase of the red fluorescence on the columella when compared with the treated Col-0 seeds. This loss of signal could be explained by the modification of the seed surface as for instance a rearrangement of the lipidic compounds over the surface due to the plasma treatment.

**Figure 5:**
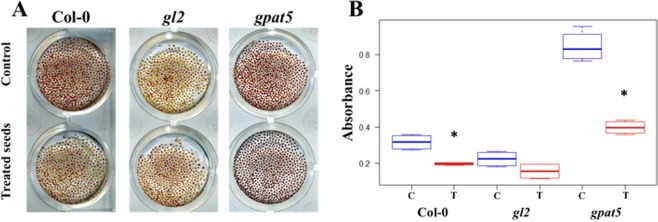
Low temperature plasma treatment modifies *A. thaliana* seed permeability. (**A**) Picture of the three Arabidopsis seed genotypes (Col-0, *gl2* and *gpat5*) treated or not with plasma. The seeds were incubated for 48 hours in tetrazolium red. (**B**) Boxplots of the absorbance of the grind seeds with tetrazolium versus the genotype and the treatment (T for plasma treatment and C for control). The blue boxplots display the control seeds and the red ones the plasma treated seeds. 3 batches of 100 µg seeds for each genotype and treatment were tested for permeability assays.

In addition to the seed surface observation, the permeability of the seeds treated or not treated with the low temperature plasma was tested with tetrazolium red. When it passes through the plant cell wall, tetrazolium red is oxidized *via* dehydrogenases in the respiratory chain and turns red. Quantification of the red staining is proportional to the seed permeability. Such tests were carried out on *A. thaliana* seeds of the 3 different lines, Col-0 which is the reference ecotype, and the two considered mutants *gl2 and gpat5*. Regardless of the genotypes, the seed permeability decreases with the low temperature plasma treatment (Fig. 5). For Col-0, the absorbance is decreased by a factor 1/3 with a p-value of 0.02857. The decrease in permeability for *gl2* seeds is not significant enough even if a small reduction was observed. The absorbance of the seeds of *gpat5* was much higher in the control condition due to the absence of cuticle and was reduced by a half after plasma treatment (from 0.8 to 0.4), with a p-value of 0.01481 at Wilcoxon test.

### Change of peroxidase activities

At least 23 CIII Prxs genes have been shown to be expressed during early germination steps (see Supplementary Fig. S2 and Supplementary Table S3 rebuilt in the present work from litterature data^47^). They were detected in both testa and endosperm tissues where the corresponding proteins could have major function to favor the rupture of the two seed envelops prior the radicle protrusion as previously demonstrated^30,48^. In this study, the whole peroxidases activity has been quantified and reported in Supplementary Fig. S2. We can see that the total peroxidases activity was increased in the case of plasma treated seeds.This is in agreement with recent studies demonstraiting the function of peroxidase in seed germination^30,48^ that could explain a part of the plasma germination activation.

## Discussion

This study highlights the partial neutralization by the plasma treatment of the adverse effects of the salt and osmotic stresses on seed germination.

Depending on the considered seed genotype and mutants, a reduction and, in some cases, an inhibition of germination in saline conditions were observed. Saline stress has negative effects largely due to the ionic imbalances^38^ and the interference in the absorption of water and essential nutrients for plant development^39,41^. In the presence of a high concentration of NaCl, the germination rate and speed strongly decrease. The seeds treated with low temperature plasma remain affected by the effects of salt stress; however, it appears that germination rate of treated seeds is still higher than the control one except for *gpat5* mutant. This means that the effect of plasma counteracts partially the negative effect of the saline stress.

If the seeds are germinated in presence of PEG, variable reduction and delay of the rate of germination are found^42^. Moreover, it has been shown that the PEG has a more negative effect than the NaCl on the germination. This could be explained by the ionic balance in the seed, the influx of NaCl will reduce the osmotic pressure but as the PEG cannot pass through the cell wall, the ionic balance remains too strong for proper water intake and then a correct seed germination^49,50^. This is in agreement with the results presented in this study. It is noted that the low temperature plasma reduces the effect of osmotic stress by allowing a better germination rate except as expected for *gpat5* mutant.

One of the hypotheses to explain the plasma-stimulated germination is an effect of limitation of exchanges with the direct environment of the seed after a plasma treatment. Indeed, it appears that the protective layers of the embryo were altered by the plasma treatment reducing the seed permeability. The decrease of the permeability due to the low temperature plasma, observed in the three genotypes, could explain the reduction of the inhibitory effect of NaCl and PEG.

To confirm this hypothesis the mutants *gl2* and *gpat5* have been used. The *GL2* gene is a transcription factor that allows the normal development of the seed coat and mucilage^22^. The *gpat5* mutant presents a lack of cuticle in the seed coat^26^. Our results confirm that the germination rates of *gl2* and *gpat5* mutants were reduced compared to the ecotype Col-0^24^. For the *gl2* mutants the observations are similar to those done for Col-0 ecotype since the low temperature plasma increases the rate and the germination speed. This can be explained in part by the weakening of the outer layers. On the other hand, the *gpat5* mutant did not germinate after the plasma treatment. As this specific *gpat5* mutant is altered in its protective layers, the underlying assumption is that seeds are more sensitive to the plasma processing and the survival of the embryo is permanently impaired. For recall, the low temperature plasmas have generally a dose-dependent effect^12,13^ since at low dose, which in this case corresponds to a shorter treatment time, this increases cell division and at high dose this inhibits it.

On the other hand, we investigated the possible effects of the low temperature plasma treatment on seed surface. The seed coat plays an important role during the germination step since it acts both as a physical protection but can also act as a constraint for the release of the radicle. As a result, any change of the outer layers can affect the seed germination. Scanning electron microscopy has clearly revealed a change on the seed surface after the plasma treatment. Thus, it appear that this surface modification can correspond to lipid compounds exuded from the seed following the plasma treatment. An increase of the germination rate and a change in the surface structure could be correlated^51^.

In addition, the modification of the surface is also confirmed by the fluorescence observations. Regardless of the Col-0 ecotype, the seed surface is similar between the different seeds without plasma treatment. After the plasma treatment, a significant modification is observed on the seed surface of Col-0 and *gl2* since a disappearance of the fluorescent signal is noted. The observation of the seeds of the *gpat5* mutants shows that the plasma has a smaller effect compared to the two other genotypes. This can be explained by the cuticle deficiency of the *gpat5* mutant and can point out the formation of a neo-lipidic structure at the seed surface after the plasma treatment.

The modification of the seed surface observed in microscopy which can be due to the change of the lipidic structure, could explain the reduction of the permeability of the plasma treated seeds compared to the controls. Plasma treatment makes the seeds less permeable which could have a beneficial effect on the physical dormancy of the seed^52^. This effect is observed for the ecotype Col-0 and the mutant *gpat5*, however we have not observed significant permeability reduction for the *gl2* seeds. But the initial low germination rate of this mutant could explain the negative effect of the plasma treatment. Complexity of fatty acid composition of the seeds but also in the signalling pathways of mantle formation could explain this observation^53^. Moreover, since the permeability of the seeds of the mutants *gl2* is initially low, it seems to make it difficult to reduce the permeability of these seeds. It is interesting to observe the significant decrease in permeability for *gpat5* seeds. Indeed, it is half reduced after the low temperature plasma treatment which clearly indicates and underlines the plasma effect without any ambiguity. This is also related to the properties of *gpat5* mutant and allows to conclude that the effect of the plasma is not only due to the alteration of the cuticle since *gpat5* does not present a synthesis of this polymer.

## Conclusion

In this study, the effects of low temperature plasmas on germination of *A. thaliana* seeds were thoroughly investigated. The low temperature plasma was generated in ambient air by a dielectric barrier discharge setup while the ecotype Col-0 and the two mutants *gl2* and *gpat5* were analyzed under osmotic and saline stresses to better understand the plasma effects on testa and endosperm ruptures during the early germination step.

It is highlighted that plasma treatment increased the germination speed and rate under the different stress conditions, which may be of great interest for agronomy. In fact, the increased germination rate of plasma-treated seeds grown in salt stress allows the investment of greater areas with saline soil. It is clearly underlined that the low temperature plasma treatment does not completely counteract the effects of stress, but it may, in some cases, compensate the germination delay. The plasma-induced changes of seed surfaces that were observed in scanning electron and confocal microscopies and the plasma–induced decrease of the seed permeability of the embryonic external layers suggests that the observed effects on seed germination are due to the changes of the seed layers. Nevertheless, the use of the ecotype Col-0 and of the two mutants *gl2* and *gpat5* illustrate the importance of the integrity of these layers. Indeed, the treatment of the mutant *gpat5* almost completely inhibits germination. This suggests that a prolonged plasma treatment or a treatment with more reactive and energetic plasma active species would have negative effects on germination.

Moreover, the present results propose that plasma treatment could affect both the changes in the structure and in the composition of lipid compounds of the seed surface prior germination and the seed metabolisms during early germination step.

## Methods

### Low temperature plasma device

Dielectric Barrier Discharge (DBD) is a device that generates low temperature plasma at atmospheric pressure between a cylindrical electrode covered with a dielectric and connected to the high voltage power supply and a grounded electrode also covered with a dielectric (Fig. 1) while ambient air is used as a background gas. Due to the possibility to use any surface as a grounded electrode, such plasma device is called floating electrode DBD (FE-DBD). The electrical parameters of the mono-polar pulsed power supply are set to 10 kV for the voltage, 10 kHz for the frequency and 1 μs for the pulse width while the rise and fall times of the voltage pulse are of about 80 ns. Further details on the plasma characteristics generated by this FE-DBD air plasma can be found elsewhere^4^.

As displayed in Fig. 1, *A. thaliana* seeds are placed in the inter-electrode gap between the glass of powered and grounded electrodes directly in contact with the air low temperature plasma. The treatment time of the different seed genotypes is fixed at 15 min which is an optimal time already parameterized and chosen in previous works^21,48^.

### Plant material

Several genotypes were used, Col-0 (which is the wild type strain carrying functional *GPAT5* and *GL2* genes) and two mutant lines (*gpat5* and *gl2*)^54,55^.

The seeds of *A. thaliana* are germinated on Whatman ™ paper circles (3MM Char Chromatography Paper, Medium thickness: 0.34 mm), which are placed in Petri dishes of 35 mm in diameter. The boxes are saddled with ANAPORE surgical plaster. *In vitro* culture conditions are considered: humidity 100%, box temperature 22 °C/20 °C, photoperiod 16 h/8 h. The paper is soaked with 0.5 mL of the following solutions: distilled water; NaCl (25 mM, 50 mM, 75 mM and 100 mM); PEG 6000 (5%, 10%, 15% and 20%) (Polyethylene glycol, BioUltra, Sigma Aldrich, Saint-Quentin Fallavier, France).

### Conduct of experiments

The *A. thaliana* seeds are treated during 15 min and placed in a Petri dish. The different seed observations are made 24 h, 40 h, 48 h and 64 h after imbibition. About 150 seeds per dish and 3 independent batches.

#### Seed germination

In order to quantify the seed germination, the rate of testa and endosperm ruptures as well as the number of cotyledons were noted using a binocular microscope. The resulting image processing is done on ImageJ version 1.46r^56^. The obtained results are entered on an Excel® table and statistically analyzed using the R studio software version 1.0.136 (cran.r-project.org). Student tests^57^ as well as Wilcoxon tests^58^ were carried out to detect the significance of the differences between the various seed analyses.

#### Seed surface

Pictures of the seed surface are made using a confocal monophoton microscope (Leica SP8). Observations are made using at least 20 seeds of the different genotypes treated or not. The optical characteristics are set as follows: excitation laser at 488 nm; reflection from 484 nm to 494 nm; auto fluorescence at 505–560 nm and 570–640 nm. The used lens is a 10 × 0.3 dry. The distance between the focal planes is fixed to 3 μm. The different seeds are prior stained with a fluorescent compound, Auramine-O at 0.003% in 70% ethanol (Sigma Aldrich). This compound allows to localize hydrophobic compounds such as the waxes and precursors of cutin as well as lipid compounds^59^.

#### Seed permeability

Seed permeability tests are performed using triphenyltetrazolium chloride (tetrazolium red, Fisher) which has the property of being reduced to formazans becoming red, as they cross the plasma membrane. The control and plasma-treated seeds are incubated with 1% tetrazolium red solution for 40 h at 28 °C. They are rinsed with distilled water and grind by hand in 95% ethanol using pestles. The grind material is centrifuged at 3500 rpm during 3 min and the absorbance of the supernatant is measured at 492 nm. Each permeability assay was performed 3 times using 10 mg of seeds from 3 independent batches of seed treated or not with the low temperature plasma.

#### Analysis of gene expression data

Gene expression data, available from seed embryogenesis and germination kinetic, were extracted from two previous studies^27,47^. The extracted data were transformed to log2 values. The gene expression limit was set up at 5, under that threshold, the expression is considered as negligible. Subsequently, *GL2* or *GPAT5* for the embryogenesis set and Peroxidase 69 (*AT5G64100*) for the germination set were used as a bait to calculate the Pearson correlation coefficient (PCC). Then, the genes are sorted and ranked according to their decreasing PCC. This allows to extract the genes that have a similar spatio-temporal expression profile. From this ranking, all the first 50 genes are selected from the embryogenesis set and only the peroxidases encoding genes were selected from the germination set. The expression profiles of the different genes are highlighted by a heatmap: gray (expression data below the threshold of 5); from yellow^60^ (minimum expression data above the threshold of 5) to red (maximum expression data).

## Supplementary information

Supplementary tables S1 S2 and S3

Supplementary figures
