# Supplementary material for: New insights of low-temperature plasma effects on germination of three genotypes of Arabidopsis thaliana seeds under osmotic and saline stresses
Source: Sci Rep. 2019 Jun 17;9:8649. doi: 10.1038/s41598-019-44927-4 (PMC6572809; doi:10.1038/s41598-019-44927-4)
Supplement: Supplementary file 2 — Supplementary figures [file 41598_2019_44927_MOESM2_ESM.pdf]

Supplementary information

Title: New insights of low-temperature plasma effects on germination of three genotypes of *Arabidopsis thaliana* seeds under osmotic and saline stresses

Authors: Maxime Bafoil, Aurélie Le Ru, Nofel Merbahi, Olivier Eichwald, Christophe Dunand, Mohammed Yousfi

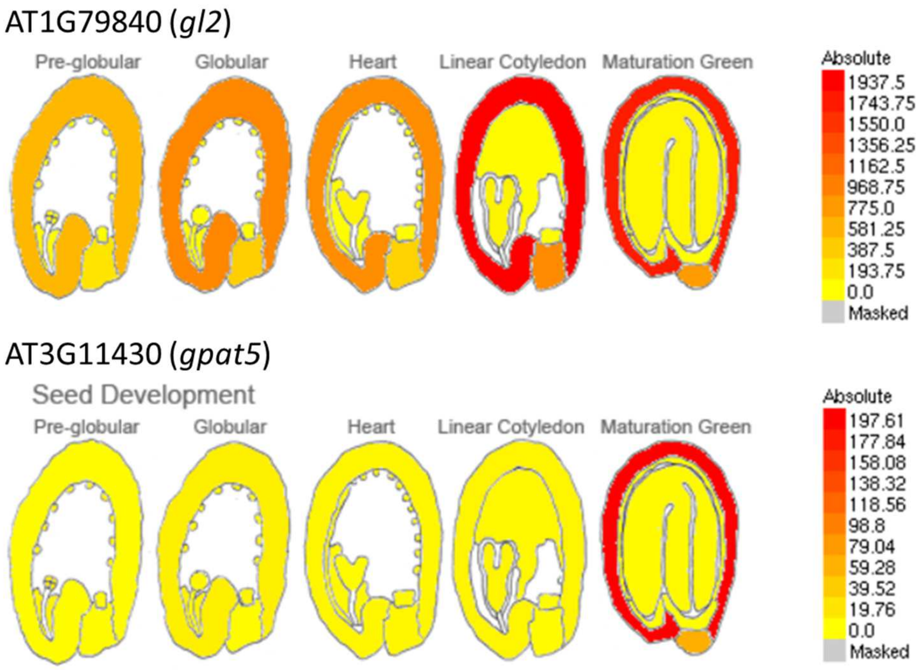

Supplementary Figure S1. Spatio-temporal expression profile of *GL2* and *GPAT5* during embryogenesis. Data have been collected from the eFP browser microstrancriptomic map (http://bbc.botany.utoronto.ca/efp/cgi-bin/efpWeb.cgi?dataSource=Seed) with absolute heatmap scale (high to low expression values corresponding to red to yellow) and adapted from Winter et al (2007) [60].

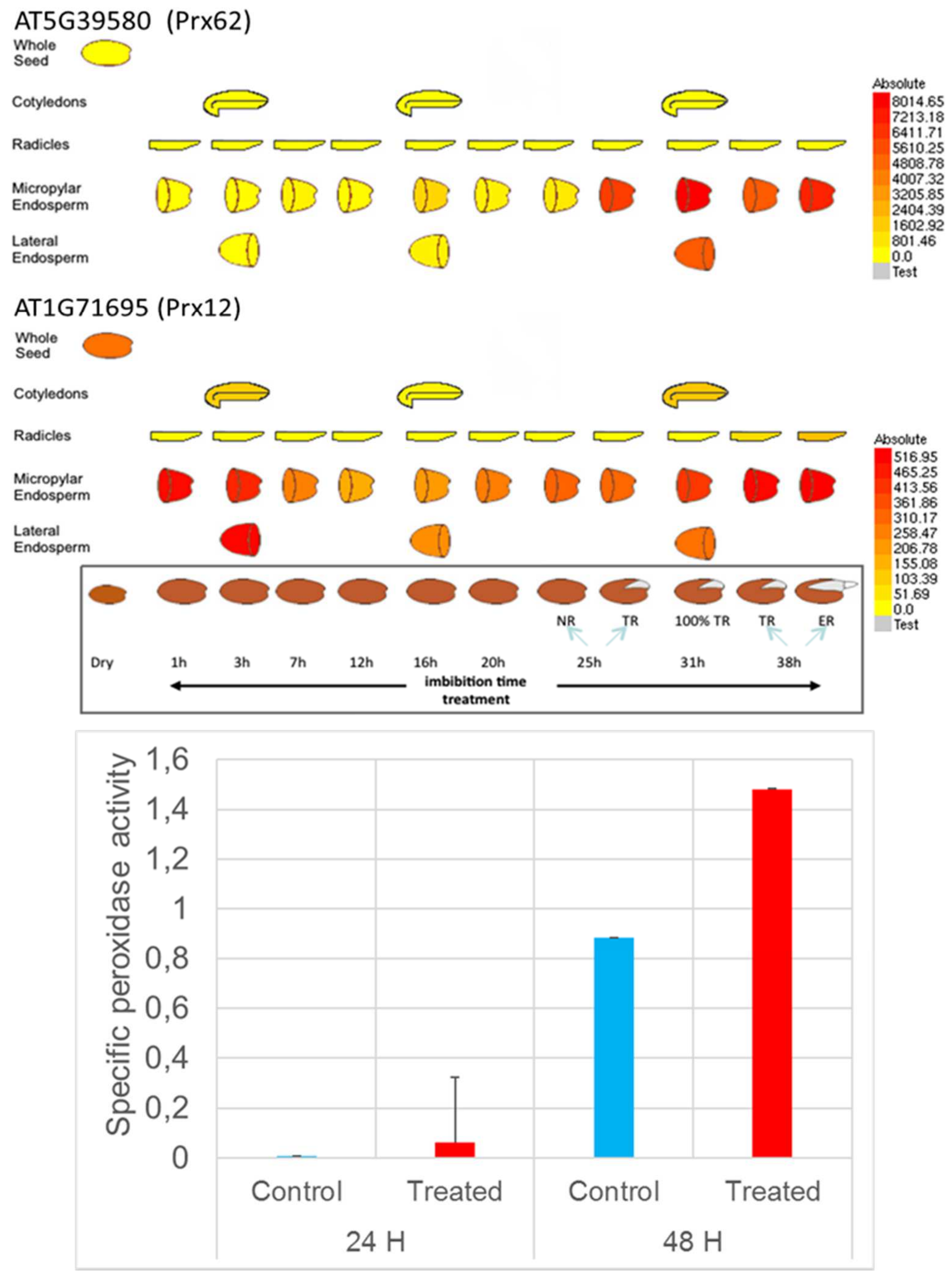

Supplementary Figure S2. Spatio-temporal profiles of two CIII Prx genes during early steps of seed germination (until 38 hours post sowing). Data have been collected from the eFP browser (http://ssbvseed01.nottingham.ac.uk/efp_browser/efpWeb.cgi) and adapted from Dekkers et al. (2013)[47]. Bar chart of specific peroxidase activity: The red bars display the plasma-treated seeds, the blue ones are the non-treated (control) seeds.

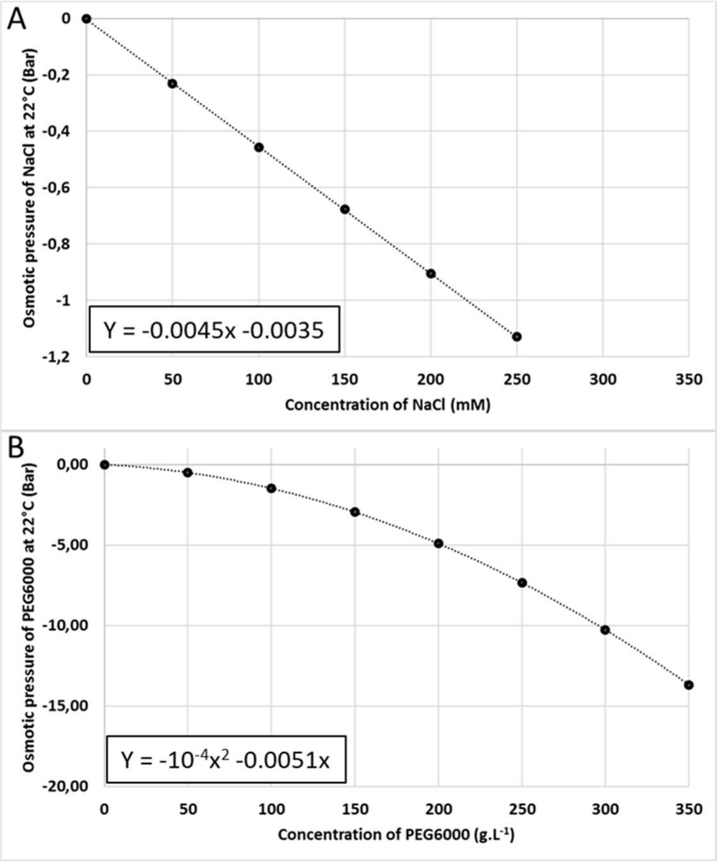

Supplementary Figure S3. Osmotic potential curves of NaCl and PEG 6000 at 22 ° C. The equation of the NaCl curve is: y= -0.0045x -0.0035; The equation of the PEG curve is: $y = -10^{-4}x^{2} - 0.0051x$. These graphs are adapted from "Effect of NaCl and PEG induced osmotic potentials on germination and early seedling growth of rice cultivars differing in salt tolerance." MZ Alam, T. Stuchbury and REL Naylor (2002)[49] "and" The osmotic potential of polyethylene glycol 6000 Michel, BE & Kaufmann, MR (1973) [43]".

Captions of supplementary tables

Title: New insights of low-temperature plasma effects on germination of three genotypes of *Arabidopsis thaliana* seeds under osmotic and saline stresses

Authors: Maxime Bafoil, Aurélie Le Ru, Nofel Merbahi, Olivier Eichwald, Christophe Dunand, Mohammed Yousfi

Supplementary Table S1. Expression of different *Arabidopsis thaliana* genes during embryogenesis. *AT1G79840* or *GL2*, which corresponds to one of the two mutant lines we used in our study, is used as a bait to calculate the Pearson Correlation Coefficient (PCC) for all *A. thaliana* genes. All genes are then ranked in descending order according to this coefficient. The first 50 genes with the most similarity to *GL2* are shown in the table. They represent all the genes which are express according to the same pattern as *GL2*. The expression values are expressed in log2. The detection threshold is set to 5 (shown in grey). A heatmap from yellow (lowest values) to red (highest values) is then applied to values above the threshold. All the values come from Belmonte et al. (2013) [23].

Supplementary Table S2. Expression of different *Arabidopsis thaliana* genes during embryogenesis. *AT3G11430* or *GPAT5*, which corresponds to one of the two mutant lines we used in our study, is used as a bait to calculate the Pearson Correlation Coefficient (PCC) for all *A. thaliana* genes. All genes are then ranked in descending order according to this coefficient. The first 50 genes with the most similarity to *GPAT5* are shown in the table. They represent all the genes which are express according to the same pattern as *GPAT5*. The expression values are expressed in log2. The detection threshold is set to 5 (shown in grey). A heatmap from yellow (lowest values) to red (highest values) is then applied to values above the threshold. All the values come from Belmonte et al. (2013) [23].

Supplementary Table S3. All data in the table have been extracted from Dekkers et al. (2013)[47]. This table represents the expression data of *Arabidopsis thaliana* genes during germination according to the different tissues considered (microdissection by laser capture). Only the lines corresponding to CIII Prxs genes were considered in this study. The data was transformed into Log2 and the detection threshold set to 5 (lower values colored in grey). On the values above the threshold, a heatmap is made by taking a yellow-red gradient corresponding to a weak-strong expression. Only the 23 CIII Prxs genes expressed during germination are retained. They are then ranked according to their maximum expression value. Legend: RAD: radicle; COT: cotyledons; MOS: micropylar endosperm and chalazal; PE: peripheral endosperm; TR: testa rupture; ER: endosperm rupture. Data from Dekkers *et al,* have been obtained from germination on culture media which faster germination speed when compare to paper filter germination conditions. Therefore, we can see a delay between the germination kinetic of Dekkers *et al.,* and our kinetics. 24 hours and 48 h in our study are the equivalent of 12-16H and 38H in Dekkers data[47]respectively.
